# Effect of foveal herniation on surgical outcomes in patients with idiopathic epiretinal membrane

**DOI:** 10.5935/0004-2749.20220047

**Published:** 2025-08-22

**Authors:** Hakan Ozdemir, Murat Karacorlu, Ahmet Elbay, Furkan Kirik

**Affiliations:** 1 Department of Ophthalmology, Faculty of Medicine, Bezmialem Vakif University, Fatih, Istanbul, Turkey; 2 Istanbul Retina Institute, Sisli, Istanbul, Turkey

**Keywords:** Epiretinal membrane, Tomography, optical coherence, Fovea centralis, Vitrectomy, Visual acuity, Membrana epirretiniana, Tomografia de coerência óptica, Fóvea central, Vitrectomia, Acuidade visual

## Abstract

**Purpose:**

This study aimed to compare the anatomical and visual outcomes of idiopathic epiretinal membrane peeling surgery, with and without foveal herniation.

**Methods:**

This retrospective, comparative, two-center study included ageand sex-matched patients exhibiting an idiopathic epiretinal membrane with and without foveal herniation (epiretinal membrane + foveal herniation group and epiretinal-membrane-only group, respectively). The baseline best-corrected visual acuity and central foveal thickness were compared within the groups through months 1, 3, 6, and 12 of follow-up postoperatively. Then, changes in these two parameters at all follow-up points were compared between the groups.

**Results:**

We enrolled 16 patients per study group. The baseline best-corrected visual acuity and central foveal thickness were not significantly different between the two groups (p>0.05). Compared with the baseline, both the best-corrected visual acuity and central foveal thickness improved significantly in both groups in all follow-ups (p<0.05), except for the best-corrected visual acuity of the epiretinal-membrane-only group after month 1 (p<0.05). The mean best-corrected visual acuity improvement after month 1 and the mean central foveal thickness reduction after months 1, 3, and 6 were significantly better in the foveal herniation + epiretinal membrane group than in the epiretinal-membrane-only group (p<0.05). However, the best-corrected visual acuity and central foveal thickness changes were not significantly different between the groups at the final visit (p>0.05).

**Conclusions:**

Although epiretinal membrane + foveal herniation demonstrated prompt anatomical and functional improvement, foveal herniation occurrence did not affect the final surgical outcomes in patients with idiopathic epiretinal membrane.

## Abstract (pt)

**Objetivo:**

Comparar os resultados anatômicos e visuais da cirurgia com peeling da membrana epirretiniana idiopática na presença e ausência de herniação foveal.

**Métodos:**

Estudo retrospectivo, comparativo, de dois centros. Pacientes com membrana epirretiniana idiopática pareados por idade e sexo com herniação foveal (grupo membrana epirretiniana + herniação foveal) e sem herniação foveal (grupo apenas com membrana epirretiniana) foram incluídos. Mudanças na acuidade visual melhor corrigida e espessura foveal central em todos os pontos de acompanhamento foram comparadas entre os grupos. A linha de base da melhor acuidade visual corrigida e a espessura foveal central foram comparadas dentro dos grupos no 1º, 3º, 6º e 12º meses de acompanhamento após a cirurgia.

**Resultados:**

Dezesseis pacientes com membrana epirretiniana + olhos com herniação foveal e 16 pacientes com olhos apenas com membrana epirretiniana foram incluídos no estudo. Não houve diferença significativa entre os grupos na linha de base com melhor acuidade visual corrigida e espessura foveal central (p>0,05), exceto para a melhor acuidade visual corrigida do grupo da membrana epirretiniana após o 1º mês (p> 0,05), a melhor acuidade visual corrigida e a espessura foveal central melhoraram significativamente em ambos os grupos em todos os acompanhamentos em comparação com a linha de base (p<0,05). A média da melhor acuidade visual corrigida melhorou após o 1º mês e a redução média da espessura foveal central após o 1º, 3º e 6º meses foram significativamente melhores no grupo de herniação foveal + membrana epirretiniana do que no grupo com apenas membrana epirretiniana (p<0,05). Não houve diferença significativa na melhor acuidade visual corrigida e nas alterações da espessura foveal central entre os grupos na visita final (p>0,05).

**Conclusões:**

Embora uma melhora anatômica e funcional bem mais precoce tenha sido mostrada no grupo membrana epirretiniana + herniação foveal, a presença de her niação foveal não afetou os resultados cirúrgicos finais em pacientes com membrana epirretiniana idiopática.

## INTRODUCTION

Epiretinal membrane (ERM) is characterized by fibrocellular proliferations on the inner retinal surface, usually in the macular region, leading to retinal ganglion cell layer or inner nuclear layer damage. ERM is caused by inner retinal layer wrinkling resulting from decreased or distorted visual acuity^(1)^. Its prevalence is 4.0%-6.2%, and it more commonly affects the elderly population^(1,2)^. According to the formation mechanisms, ERMs are divided into two types: idiopathic and secondary. Idiopathic ERM, which is more common, develops without any underlying ocular disease. Meanwhile, secondary ERM is associated with various intraocular conditions, including trauma, retinal detachment, and retinal vascular diseases^(2)^. Generally, ERM progresses quite slowly, with some patients complaining of reduced visual acuity and/ or visual distortion that may require treatment. Currently, ERM is solely treated by surgically removing the membrane to improve visual function. Surgery without faults or complications can achieve the desired structural healing; however, some patients remain dissatisfied with the visual outcomes^(3,4)^.

ERM is successfully diagnosed by optical coherence tomography (OCT), which is a fast, noninvasive, and high-res olution retinal imaging method. OCT can identify various ERM-induced retinal changes, such as lamellar macular or pseudomacular holes, foveoschisis, disorga nization of the retinal inner layers (DRIL), disrupted outer retinal layers, ectopic inner foveal layers, and in creased macular thickness^(5-10)^. Recently, the effect of retinal changes caused by ERM on visual prognosis has been extensively evaluated. Various prognostic factors (PFs) and biomarkers related to final visual acuity have been identified after the ERM peeling surgery^(8,11-14)^. Foveal herniation (FH) is one of the rare typical tissue alterations observed with ERM. FH is defined as the herniation of the inner retinal layers through the ERM opening in the foveal region into the vitreous cavity^(5,15)^. However, only one study has investigated the effects of FH on surgical outcomes^(16)^, but this noncomparative study insufficiently demonstrated the clinical and prognostic significance of FH. To our knowledge, no comparative study has been conducted to evaluate the effect of FH on anatomical and functional outcomes in patients diagnosed with ERM after ERM peeling surgery.

This study aimed to compare the anatomical and visual outcomes of ERM peeling surgery in the presence and absence of FH in patients with idiopathic ERM.

## METHODS

### Study design and population

All patients who underwent pars plana vitrectomy (PPV) for ERM peeling at Bezmialem Vakif University and Istanbul Retina Institute between September 2014 and June 2017 were retrospectively reviewed. The inclusion criteria were as follows: the presence of FH with idiopathic ERM diagnosed by OCT; no evidence of ocular diseases that may cause macular edema or ERM (e.g., diabetic retinopathy, retinal vein occlusion, uveitis, or vitreomacular traction); no evidence of glaucoma or ocular hypertension; no history of intraocular surgery other than uncomplicated phacoemulsification surgery and ERM peeling surgery; and a follow-up period of ≥1 year after surgery. Patients meeting such criteria were classified as the ERM + FH group. In addition, ageand sex-matched patients with ERM who met the abovementioned criteria with the exception of FH occurrence were included as the ERM-only group. Conversely, we excluded patients who had received intravitreal triamcinolone and/ or anti-vascular endothelial growth factor treatment, had undergone retinal photocoagulation, and/or had visually significant cataracts or other eye disturbances that prevented a detailed fundus examination.

This study conformed to the principles of the Declaration of Helsinki and was approved by the ethics committee of Bezmialem Vakif University (June 26, 2018; No. 14/95). All participants provided informed consent.

### Ophthalmological examination and retinal imaging

After obtaining a detailed ophthalmic and medical history of the patients, we conducted a comprehensive ophthalmic examination, which included best-corrected visual acuity (BCVA) measurement with a Snellen chart, slit lamp biomicroscopy, lens status and indirect ophthalmoscopy with a 90-diopter precorneal lens, and specral domain-OCT imaging. According to The Lens Opacities Classification System III (LCOS III) criteria^(17)^, lens opacification was classified as mild, moderate, or severe, as previously described^(18)^.

OCT imaging results were examined using Spectral domain-OCT (Heidelberg Engineering, Heidelberg, Germany) with standard Spectral domain-OCT scans (512 A-scans, 20 × 15°). FH was defined as the herniation of the superficial layers of the retina toward the vitreous space via the ERM opening. The length between the inner retinal and outer retinal surfaces at the fovea centralis indicated the central foveal thickness (CFT). Baseline parameters included the BCVA and CFT measurement.

### Surgical technique and follow-up protocol

**Figure 1:**
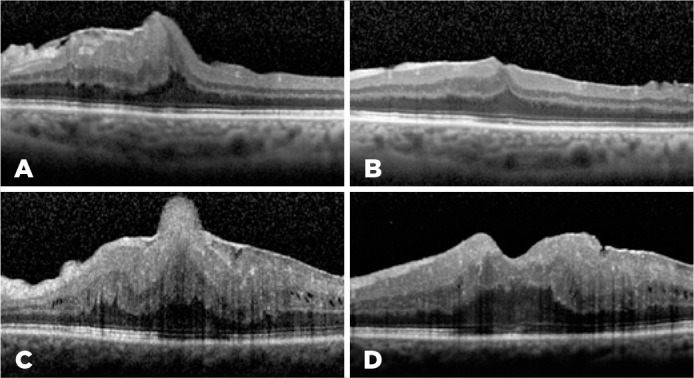
Horizontal cross-sectional optical coherence tomography ima ges of two cases before the membrane peeling surgery (a, c) and after the surgery at the final follow-up examination (b, d).

One of two surgeons (HO or MK) performed the operations using a 23-gage transconjunctival PPV with membrane peeling under sterile conditions in an operating room. All patients with mildly graded cataracts underwent phacoemulsification and intraocular lens implantation. Before peeling, ERM was stained with trypan blue solution (0.06%). Intraoperatively, surgeons planned to preserve the internal limiting membrane of all patients. The surgical procedure was completed without tamponade requirement. Postoperatively, ofloxacin (0.3%) and dexamethasone (0.1%) eye drops were prescribed six times a day for 2 weeks. Patients were examined on postoperative days 1 and 7. Thereafter, patients were re-examined by measuring the BCVA and CFT after months 1, 3, 6, and 12. Figure 1 shows the horizontal cross-sectional OCT images of two cases preoperatively (a, c) and at the final examination postoperatively (b, d).

### Statistical analysis

All numerical data are expressed as mean ± standard deviation (SD). All categorical variables are expressed as number and percentage (n, %). Normality was initially assessed using the Kolmogorov-Smirnov test. The BCVA and CFT were compared between the baseline and follow-up points in each group by using the paired *t* test for normally distributed data and Wilcoxon signed-rank test for non-normally distributed data. For comparing the mean changes in BCVA and CFT between the groups at all follow-up points, we used the independent-samples *t*-test for normally distributed data and Mann-Whitney *U* test for non-normally distributed data. Categorical data such as lens status and surgical procedure type were compared between the groups by using the Pearson’s chi-square test or Fisher’s exact test. Furthermore, statistical significance was set at p≤0.05.

## RESULTS

### Baseline data

Of the 383 eyes that underwent ERM peeling surgery, 16 (4.18%) eyes with FH were included in the study. For the control group, we included 16 ageand sex-matched eyes without FH (ERM-only). Each group had nine (56.35%) females and seven (43.7%) males. The mean age was 67.88 ± 4.13 years in the ERM + FH group and 68.06 ± 4.51 years in the ERM-only group. Age and sex were not significantly different between the groups (p=0.903 and p=1.00, respectively). Preoperatively, the ellipsoid zone (EZ) was viewed in three of the 16 eyes in the ERM + FH group and four of the 16 eyes in the ERM-only group. At baseline, the mean BCVA was 0.47 ± 0.22 and 0.57 ± 0.21 logMAR in the ERM + FH and ERM-only groups, respectively, with no significant difference (p=0.166). Meanwhile, the mean CFT at baseline was 665.06 ± 140.51 and 626.75 ± 82.76 µm in the ERM + FH and ERM-only groups, respectively, and no significant difference was also observed (p=0.677). Table 1 summarizes the demographic and baseline ocular characteristics of the two groups.

**Table 1:** Comparison of demographic data, baseline values, the mean changes in BCVA and CFT, lens status, and surgical procedure type between the groups

	ERM + FH (n=16)	ERM-only (n=16)	p value
Age, mean ± SD	67.66 ± 8.67	68.06 ± 4.51	0.903^*^
Gender, %			1.000^†^
Female	56.3	56.3	
Male	43.7	43.7	
Baseline BCVA ± SD *logMAR*	0.47 ± 0.22	0.57 ± 0.21	0.166^‡^
Changes in BCVA ± SD *logMAR* Month 1	0.10 ± 0.13	0.01 ± 0.05	**0.017** ^‡^
Month 3	0.13 ± 0.16	0.12 ± 0.11	0.91^‡^
Month 6	0.20 ± 0.16	0.22 ± 0.13	0.52^‡^
Month 12	0.26 ± 0.16	0.27 ± 0.13	0.84^‡^
Baseline CFT ± SD, µ*m*	665.06 ± 140.51	626.75 ± 82.76	0.677^*^
Changes in CFT ± SD, µ*m* Month 1	240.50 ± 133.92	42.31 ± 50.27	**<0.001** ^‡^
Month 3	281.38 ± 131.35	107.00 ± 54.64	<0.001^*^
Month 6	306.94 ± 133.88	195.81 ± 55.46	**0.002** ^‡^
Month 12	321.81 ± 124.63	267.75 ± 54.35	0.293^‡^
Lens status at baseline, n (pseudophakic-phakic)	9-7	10-6	0.719^†^
Surgical approach, n (Only PPV-Phaco + PPV)	12-4	13-3	0.674^†^
Lens status at final visit, n (pseudophakic-phakic)	15-1	15-1	1.000^†^

BCVA= best-corrected visual acuity; logMAR= log of the Minimum Angle of Resolution; SD= standard deviation; CFT= central foveal thickness; ERM + FH= epiretinal membrane with foveal herniation; ERM-only= epiretinal membrane without foveal herniation; PPV= pars plana vitrectomy; Phaco + PPV= phacoemulsification surgery combined with pars plana vitrectomy.

* Independent *t*-test.

† Chi-square test.

‡ Mann-Whitney *U* test.Values with p<0.05 are shown in bold.

### Comparison of BCVA and CFT changes between the groups

Table 1 summarizes the comparison of BCVA and CFT changes between the groups at all follow-ups.

At months 1, 3, 6, and 12 of follow-up, the mean BCVA changes were 0.10 ± 0.13, 0.13 ± 0.16, 0.20 ± 0.16, and 0.26 ± 0.16 logMAR in the ERM + FH group and 0.01 ± 0.05, 0.12 ± 0.11, 0.22 ± 0.13, and 0.27 ± 0.13 logMAR in the ERM-only group, respectively. Although the mean BCVA change at month 1 was statistically better in the ERM + FH group than in the ERM-only group, no significant differences were observed between them at the other follow-up points (p=0.017, p=0.91, p=0.52, and p=0.84, respectively).

Moreover, the mean CFT changes at months 1, 3, 6, and 12 were 240.50 ± 133.92, 281.38 ± 131.35, 306.94 ± 133.88, and 321.81 ± 124.63 µm in the ERM + FH group and 42.31 ± 50.27, 107.00 ± 54.64, 195.81 ± 55.46, and 267.75 ± 54.35 µm in the ERM-only group, respectively. Although the changes were significantly higher in the ERM + FH group after months 1, 3, and 6 than in the ERM-only group, no significant difference was observed after month 12 (p<0.001, p<0.001, p=0.002, and p=0.239, respectively).

### Comparison between BCVA and CFT changes and the baseline during follow-ups

Table 2 summarizes the comparison between the baseline BCVA and CFT and their postoperative changes during follow-ups in each group. In the ERM + FH group, the mean BCVAs at months 1, 3, 6, and 12 of follow-up were 0.37 ± 0.14, 0.34 ± 0.15, 0.27 ± 0.09, and 0.21 ± 0.10 logMAR, respectively; subsequently, BCVA significantly improved in all follow-up visits (p=0.011, p=0.007, p=0.001, and p=0.001, respectively). In the ERM-only group, the mean BCVAs at months 1, 3, 6, and 12 of follow-up were 0.56 ± 0.21, 0.45 ± 0.13, 0.35 ± 0.12, and 0.30 ± 0.14 logMAR, respectively; although the BCVA at month 1 of follow-up was not significantly different from the baseline, those at months 3, 6, and 12 showed significant improvement (p=0.317, p=0.003, p<0.001, and p<0.001, respectively).

**Table 2:** Comparison of baseline BCVA and CFT with postoperative follow-ups in each group

			Baseline	Month 1	Month 3	Month 6	Month 12	
BCVA (logMAR) mean ± SD p value		*ERM + FH*	0.47 ± 0.22	0.37 ± 0.14 p=0.011^*^	0.34 ± 0.15 p=0.007^*^	0.27 ± 0.09 p=0.001^*^	0.21 ± 0.10 p=0.001^*^	
		*ERM-only*	0.57 ± 0.21	0.56 ± 0.21	0.45 ± 0.13	0.35 ± 0.12	0.30 ± 0.14	
				p=0.317^†^	**p=0.003** ^†^	**p<0.001** ^†^	**p<0.001** ^†^	
	CFT (µm) p value	*ERM + FH*	665.06 ± 140.51	424.56 ± 65.06	383.69 ± 57.76	358.13 ± 42.27	343.25 ± 51.64	
				p<0.001^*^	p<0.001^*^	p<0.001^*^	p<0.001^*^	
		*ERM-only*	626.75 ± 82.76	584.44 ± 101.36	519.75 ± 89.81	430.94 ± 68.20	359.00 ± 50.25	
				p=0.004^*^	p<0.001^*^	p<0.001^*^	p<0.001^*^	

BCVA= best-corrected visual acuity; logMAR= log of the Minimum Angle of Resolution; SD= standard deviation; CFT= central foveal thickness; ERM + FH= epiretinal membrane with foveal herniation; ERM-only= epiretinal membrane without foveal herniation.

* Paired *t*-test.

† Wilcoxon signed-rank test.

Values with p<0.05 are shown in bold.

**Graph 1:**
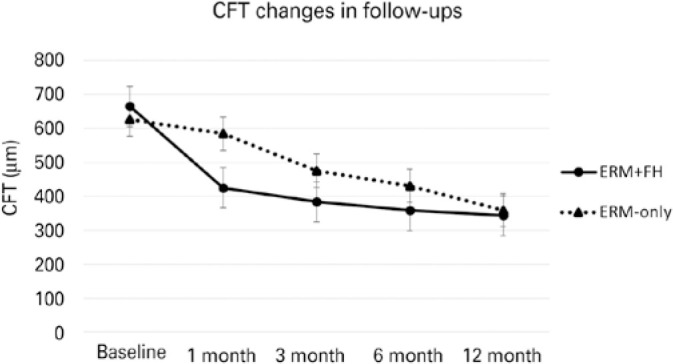
Graph showing the mean central foveal thickness (CFT) of the epiretinal membrane (ERM) with foveal herniation (FH) (ERM + FH) group and the ERM without FH (ERM-only) group during follow-ups.

Meanwhile, the mean CFT in the ERM + FH group was 424.56 ± 65.06, 383.69 ± 57.76, 358.13 ± 42.27, and 343.25 ± 51.64 µm after months 1, 3, 6, and 12, respectively, showing a significant decrease in all follow-ups compared with the baseline (p>0.05, for all comparisons). In the ERM-only group, the mean CFT after months 1, 3, 6, and 12 was 584.44 ± 101.36, 519.75 ± 89.81, 430.94 ± 68.20, and 359.00 ± 50.25 µm, respectively, also demonstrating a significant decrease in all follow-ups compared with the baseline (p=0.004; p<0.001; p<0.001; p<0.001, respectively). These details are summarized in graph 1.

### Comparison of lens status between the groups

In the ERM + FH group, 9 (56.3%) eyes were pseudophakic, while 7 (43.7%) eyes were phakic at baseline. During the follow-up period, 4 (25%) phakic eyes with mild cataracts underwent phacoemulsification surgery with PPV, and 2 of 3 (12.5%) phakic eyes with mild cataract formation underwent phacoemulsification surgery. In the ERM-only group, 10 (62.5%) eyes were pseudophakic, while 6 (37.5%) eyes were phakic at baseline. During the follow-up period, 3 (18.8%) phakic eyes with mild cataracts underwent phacoemulsification surgery with PPV, and 2 of 3 (12.5%) phakic eyes with mild cataract formation underwent phacoemulsification surgery. Lens status was significantly different between the groups (Table 1). Furthermore, no complications related to PPV or phacoemulsification surgery were observed during follow-ups.

## DISCUSSION

This study suggests that FH accompanied with ERM is not a poor PF for surgical outcomes such as BCVA and CFT. Moreover, in similar demographic and ocular conditions, visual and anatomical recovery time of eyes with FH may be faster than that of eyes with ERM but no FH.

FH rarely occurs in patients with ERM. In retinal examination, the foveal region has a bulge with distinct borders resembling a pseudohole, and OCT reveals herniation of superficial retina layers into the vitreous space through the ERM opening at the foveal region^(5,15)^. In FH cases, CFT increases. Thus, FH may have an impact on surgical outcomes^(12)^.

Only one study focusing on FH has ever evaluated the surgical outcomes of ERM peeling surgery in patients with ERM + FH^(16)^. In this noncomparative study, the foveal contour, CFT, and BCVA of 11 patients with ERM + FH were evaluated retrospectively for at least 12 months, and CFT and BCVA improved at all follow-up points compared with the baseline, consistent with the present study. However, the clinical importance of FH cannot be adequately explained by this noncomparative study. Their study results were relatively different from ours. While the change in BCVA was approximately 0.26 ± 0.16 logMAR at year 1 of follow-up in the present study, it was approximately 1.2 logMAR in the previous study. Additionally, contrary to the continuous decrease in CFT, BCVA was generally stable after 1 month postoperatively in the previous study (0.49 ± 0.12 and 0.49 ± 0.17 logMAR at month 1 of follow-up and at the final follow-up, respectively). These differences between the results of the two studies can be explained by the idea that the initial BCVA of the other study was worse than that of the present study and poor preoperative BCVA indicates a poor PF (0.61 ± 0.16 logMAR vs. 0.47 ± 0.22 logMAR).

Other postoperative PFs of ERM peeling surgery have been extensively explored. Age, preoperative symptom duration, preoperative BCVA, and preoperative metamorphopsia were suggested as PFs^(12)^. Kauffmann et al. reported that the outcome can be worse if symptoms persists for >12 months compared with <12 months, but their reports about baseline VA are inconsistent^(19)^. When Spectral domain-OCT became available, microstructural factors were suggested as factors influencing the outcome. Retina and choroid deformation quantification and various microstructural indices were also investigated^(3)^. OCT studies showed that EZ disruption, DRIL presence, cone outer segment tip line deterioration, cystic macular edema, and thick CFT were poor PFs, while photoreceptor outer segment length was a good PF^(3,12-14,20-22)^. In the present study, EZ was viewed in only 3 of the 16 eyes in the ERM + FH group and in 4 of the 16 eyes in the ERM-only group. The inability to visualize EZ was caused by the shadowing effect; thus, we could not confirm EZ disruption as a poor PF. Likewise, the presence of DRIL, which is a prognostic biomarker, could not be evaluated between the groups. As described, the FH is the protrusion of the inner retinal layers from the ERM toward the vitreous space^(15)^. Though the pathophysiology of FH remains unknown, the laminar anatomical structure of the retina is expected to be disrupted, particularly in the inner retinal layers. Hence, evaluating the presence of DRIL, which indicates poor surgical outcome in eyes with idiopathic ERM, might lead to erroneous interpretations in eyes with FH^(8)^.

As mentioned, patient age and preoperative BCVA and CFT are PFs in patients with ERM. In the current study, both ERM groups were ageand sex-matched, and baseline BCVA and CFT showed no significant diffe rences between the groups. Thus, with the similarity of these demographic data between these groups, the effect of FH on surgical outcomes was evaluated, with the variable least likely to affect the prognosis.

Our study, however, has some limitations. For instance, patient records of metamorphopsia and visual symptom duration were lacking. Considering the rarity of FH, the sample size was small; hence, prospective studies with larger series are needed. Another limitation is that the presence of phakic patients at the beginning of our study may have potentially affected the results because preoperative cataract and postoperative cataract risk were both present in this study. However, patients who underwent lens extraction had mild cataracts, and those with clear lens did not develop cataract during the follow-ups. As such, this potential effect may be neglected.

In conclusion, vitrectomy and ERM stripping surgery obtained successful outcomes in FH-associated ERM cases. FH had no poor effect on long-term surgical outcomes in patients with ERM. Additional studies with a larger sample size may further elucidate the effect of FH on ERM prognosis.
